# Orbital cellulitis with posterior scleritis in a 13-year-old girl: A case report

**DOI:** 10.1097/MD.0000000000047410

**Published:** 2026-01-30

**Authors:** Lei Yang, Shuangle Li

**Affiliations:** a Department of Ophthalmology, Zigong First People’s Hospital, Zigong, China.

**Keywords:** exudative retinal detachmen, optic neuropathy, orbital cellulitis, posterior scleritis

## Abstract

**Rationale:**

Clinical signs and symptoms of posterior scleritis vary and are easily confused with other diseases. Inflammation spreads anteriorly, involving the upper lid structures, causing lid swelling and simulating cellulitis. However, we report a case of orbital cellulitis with posterior scleritis and complications, with the aim of helping clinicians identify and treat early to prevent irreversible loss of vision.

**Patient concerns:**

A 13-year-old girl presented with pain in her right eye and an acute deterioration in vision.

**Diagnoses:**

The case was diagnosed as orbital cellulitis with posterior scleritis. Investigations revealed a raised C-reactive protein, erythrocyte sedimentation rate, and white cell count. Fundus examination revealed exudative retinal detachment, optic disc, and macular edema with a T-sign on B-scan ultrasound. Computed tomography of the paranasal sinuses and orbits reveals sinusitis and orbital inflammation.

**Interventions:**

Significant regression was observed in ocular symptoms and signs with antibiotic and steroid therapy.

**Outcomes:**

After 2 months, her vision improved significantly, but the baseline was still not reached.

**Lessons:**

Orbital cellulitis and posterior scleritis are often used as a differential diagnoses. However, this case showed that orbital cellulitis and posterior scleritis can occur simultaneously, especially in the presence of some obvious cause for or indicators of infection.

## 1. Introduction

Clinical signs and symptoms of posterior scleritis are varied and easily confused with those of other diseases, and inflammation spreads anteriorly, involving the upper lid structures, causing lid swelling, and simulating cellulitis. Previous reports of cases mimicking orbital cellulitis have presented with conjunctival injection and elevated inflammatory indicators, without sinusitis or orbital inflammation.^[1–3]^ B-mode ultrasonography showed scleral thickening and a “T” sign. All patients in their cases responded well to oral steroids. Therefore, they preferred the single diagnosis of posterior scleritis. However, we report a case of orbital cellulitis with posterior scleritis and complications such as optic disc, macular edema and exudative retinal detachment (ERD). The patient responded well to steroids and antibiotics. The key is that she had sinusitis, sinonasal symptoms, and orbital inflammation.

## 2. Clinical record

**Figure 1.**
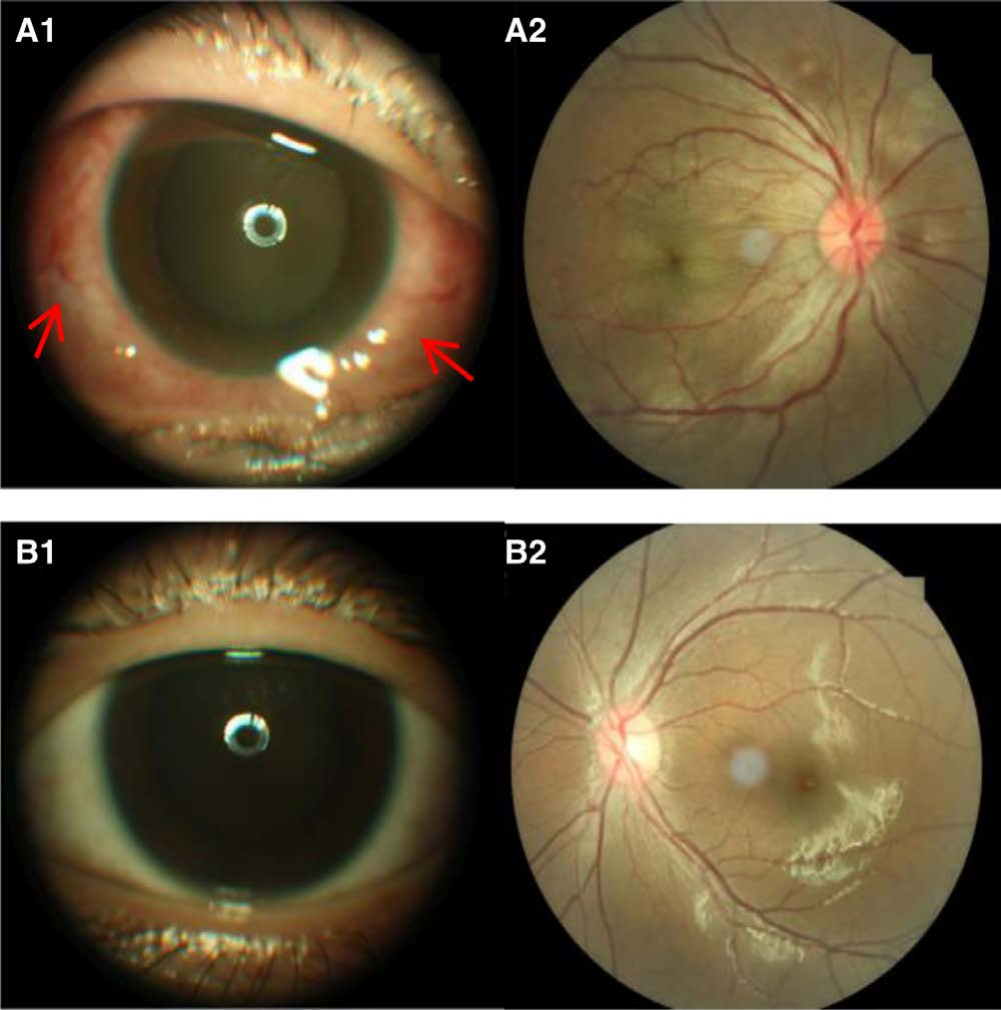
Anterior segment and fundus of both eyes on admission. (A1) The right eye showing upper lid erythema, edema, and conjunctival injection with chemosis. (A2) Optic disc edema with blurred margins, tortuous vessels, and macular edema. (B1–B2) Anterior segment and normal fundus in the left eye.

**Figure 2.**
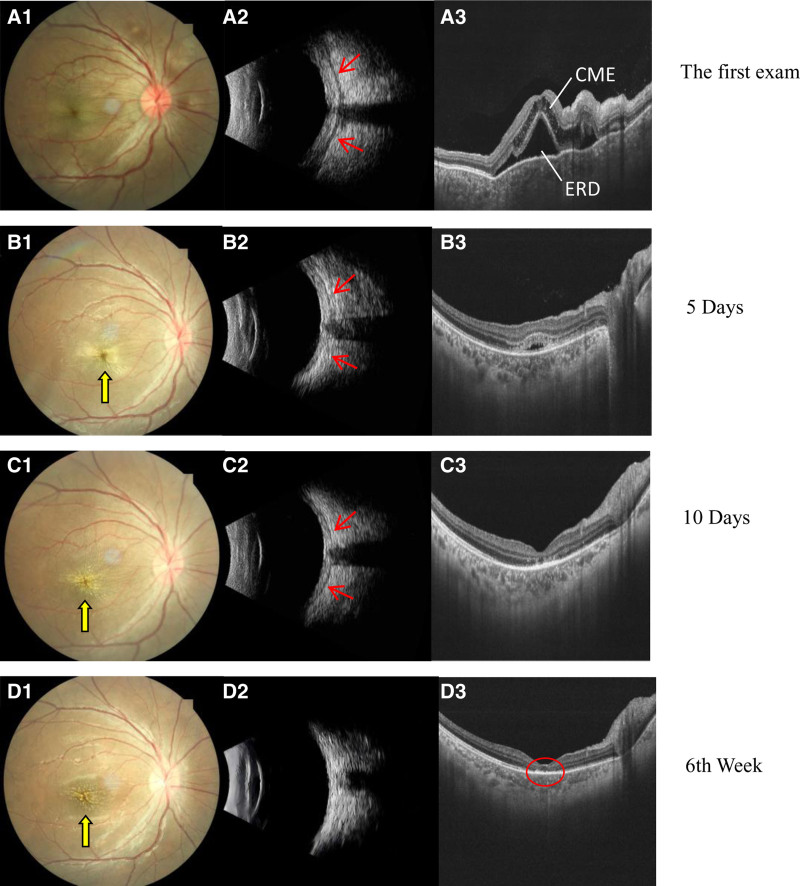
Fundus, B-scan ultrasound, and optical coherence tomography on admission, and after 5-day, 10-day, and 6-week therapy in the right eye. (A1) Right fundus showing disc edema and tortuous vessels. (B1, C1) Subretinal exudates at the macula occurring during treatment. (D1) Fundus exudates remaining at the macula after 6-week therapy. (A2) B-scan ultrasound of the right eye showing a prominent T-sign. (B2, C2) The eye wall gradually thinning after therapy. (D2) Resolution of the T-sign. (A3) Optical coherence tomography showing cystoid macular edema (CME) and exudative retinal detachment (ERD) in the right eye. (B3, C3) The macular edema is reducd, and the subretinal fluid is absorbed after therapy. (D3) Retinal structure disordering, ellipsoid zone and the interdigitation zone continuity interruption.

**Figure 3.**
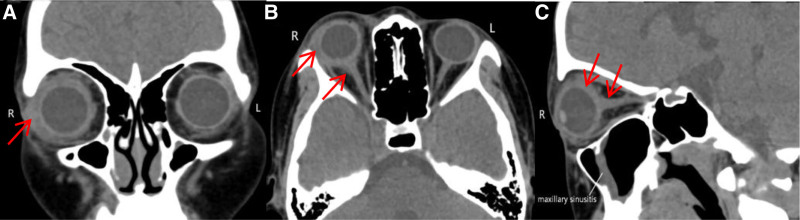
Computed tomography scans (coronal, A; axial, B; and sagittal views, C) of the same patient demonstrating evidence of the right maxillary sinusitis, marked thickening of the right eye and as optic nerve along its orbital track.

A 13-year-old girl presented to our clinic with a 7-day history of a painful right eye associated with periorbital swelling and an acute deterioration in vision. The patient had no history of injury or sty. She developed sinonasal symptoms such as nasal obstruction and rhinorrhea before onset. At presentation, the patient’s temperature rose to 37.5°C. Her right eye visual acuity was 0.04 (no improvement was achieved after correction) and the best-corrected visual acuity (BCVA) in the left eye was 1.0. Intraocular pressure was normal. All gaze directions of the right eye, especially abduction and supraduction, show slight to moderate limitation of ocular movement, which may be related to the patient’s ocular pain and unwillingness to turn the eye. Computed tomography scans (CT) measurements showed a 2 mm right eye exophthalmos (compared to the left eye; CT measuring method: on the CT axial image, using the baseline formed by the line connecting the bony orbital margin vertices, measure the perpendicular distance from the corneal vertex to this baseline). The right eye showed upper lid erythema, edema, and conjunctival injection with chemosis (Fig. 1.A1). Optic disc edema with blurred margins, tortuous vessels, and macular edema were observed on fundus examination (Fig. 1.A2). Relative afferent pupillary defects were positive. Color vision decreased. Biomicroscopic examination revealed normal anterior segment findings and fundus examination of the left eye (Fig. 1.B1, B2). Investigations revealed a raised C-reactive protein (CRP) (8.45 mg/L), raised erythrocyte sedimentation rate (ESR) (70 mm/h), and raised white cell count (15.09 × 10^9^/L). Thyroid, liver, and renal function test results were normal. A full autoimmune blood screening test was performed, which yielded negative results. Optical coherence tomography (OCT) revealed cystoid macular edema, ERD at the posterior pole and retinal folds in the right eye (Fig. 2.A3). B-scan ultrasonography showed classic T-sign and marked thickening of the eye wall (Fig. 2.A2). CT of the orbits and sinuses revealed maxillary sinusitis, excluding extraocular muscle enlargement, subperiosteal abscess, or collection. Crucially, the lesions of the orbit were not limited to the inflammation of the eyelid, but also included the blurred fat gap of the orbit, the thickening of the scleral wall and optic nerve, indicating the coexistence of orbital cellulitis and posterior scleritis (Fig. 3). We agree that severe posterior scleritis itself can cause periorbital inflammation. However, the CT findings in this case, which revealed extensive infiltration of orbital fat and thickening of the optic nerve sheath, combined with definitive evidence of sinusitis, strongly support the presence of an independent or secondary infectious inflammatory component in the orbit.

Based on these findings, a provisional diagnosis of orbital cellulitis with posterior scleritis was made. She was admitted and initiated intravenous broad-spectrum antibiotics immediately and empirical treatment for periorbital cellulitis. No pathogenic bacteria were detected in the blood cultures. After 24 hours of initiating antibiotic therapy, given the severity of the inflammatory response and concerns for visual prognosis, she was started on intravenous methylprednisolone (500 mg/d for 3 consecutive days). The patient was started on oral prednisone (1 mg/kg body weight) on the 4th day, and was weaned off by 5 mg/wk. The patient’s pain and exudative detachment were relieved 5 days after the initiation of treatment. Star-like exudation appeared in the macular area, with an improvement of visual acuity to 0.08 (Fig. 2.B1). The ESR and CRP level and blood leukocyte count returned to normal. Complete recovery of ERD was evident about 10 days later, and the visual acuity improved to 0.1. B-scan ultrasonography still revealed fluid in Tenon’s capsule and scleral thickening (Fig. 2.C2).

The condition of the right eye was stabilized, and the patient was discharged. However, she did not continue oral prednisone therapy. One week after discharge, the patient represented to the emergency department with recurrence of right eye pain, visual acuity reduction and discomfort. Investigations during the second admission revealed normal CRP levels and a normal blood leukocyte count. B-scan ultrasonography showing classic T-sign and marked thickening of the posterior eye wall. The fundus showed optic disc edema. OCT showed exudative macular detachment. This finding is consistent with a previous diagnosis of posterior scleritis. Consequently, steroid therapy was reinitiated and a more prolonged, slower taper was adopted. However, the patient refused long-term steroid therapy because of weight gain. Nonsteroidal anti-inflammatory treatment with indomethacin (at a dose of 25 mg 2× daily, orally) was introduced. At a follow-up clinic appointment 6 weeks after discharge, she was asymptomatic, and her vision improved to 0.3.

## 3. Discussion

The simultaneous occurrence of orbital cellulitis and posterior scleritis presents a unique diagnostic challenge due to the overlap of clinical manifestations such as pain, erythema, and potential vision loss. This challenge is compounded by the broad differential diagnosis, which includes orbital pseudotumor, infectious endophthalmitis, Vogt–Koyanagi–Harada (VKH) spectrum disease, and optic neuritis. Orbital pseudotumor can closely resemble an acute posterior scleritis, both of which may cause scleral thickening, retrobulbar edema, and extraocular muscle hypertrophy. Inflammatory pseudotumor may cause scleritis, although it is uncommon.^[4]^ However, in posterior scleritis, intraocular manifestations such as retinal and optic nerve involvement are more prominent than extraocular symptoms. The diagnosis of inflammatory pseudotumor is typically based on ultrasonographic or CT scans revealing orbital masses. Infectious endophthalmitis is acute and progresses rapidly, usually caused by eye trauma, corneal ulcer, intraocular surgery, etc. Infectious endophthalmitis was deemed less likely in the absence of recent trauma or surgery and with a clear focus of sinusitis. Although VKH is uncommon in this age group, it should still be considered given the presence of ERD. In this case, the key distinguishing feature lies in the characteristic manifestations of OCT. Compared to VKH, posterior scleritis typically exhibits a single rather than multiple ERDs on OCT (as seen in this case), and is less likely to present with the multiple folds or elevations of the retinal pigment epithelium commonly observed in VKH.^[5]^ While a few cases of VKH with unilateral clinical manifestations have been reported,^[6,7]^ bilateral ocular involvement is considered essential for the diagnosis of VKH.^[8]^ Most cases of optic neuritis are acute onset, with possible upper respiratory tract infection before onset. Typical features include severe vision loss and prominent optic disc edema. However, the sclera is rarely involved and the typical “T” sign of scleritis is not seen on B-ultrasound.

Orbital cellulitis has a rapid onset, often causing ocular conjunctival edema and periorbital swelling, along with limited eye movement, eye protrusion, and sometimes vision loss.^[9]^ It can even lead to life-threatening intracranial complications or sepsis. The most common cause is sinusitis, which spreads to the eye. Therefore, prompt anti-infective treatment is required. Given the severity of visual impairment and evidence of significant clinical/imaging inflammation, we administered methylprednisolone intravenously. This decision was based on acute and severe visual threat, primarily driven by an invasive inflammatory process. The combination of systemic glucocorticoids with systemic antibiotic therapy for orbital cellulitis reduces the risk of orbital inflammation and is associated with a lower likelihood of infection exacerbation. An early combination of steroids can help shorten hospitalization and prevent the progression of inflammation.^[10]^

Posterior scleritis is difficult to diagnose and more likely to be misdiagnosed. Pritam et al reported 3 cases of posterior scleritis associated with optic neuropathy and ERD previously misdiagnosed as a range of conditions, such as Pseudotumor and Papillitis.^[11]^ In all cases, clinical resolution occurred with the use of systemic steroids. Posterior scleritis is rarer in children than in adult population.^[12]^ The presence of the T-sign is a typical sign of sclerosis. However, not all cases of sclero show a typical “T” sign on B-scan ultrasonography.^[5]^ In particular, scleritis is difficult to differentiate from orbital cellulitis when it presents with protruding eyeballs, eyelid swelling, redness, pain, limited eye movement, and acute vision loss. According to our investigation, 3 cases of scleritis masquerading as orbital cellulitis have been reported.^[1–3]^ None of the patients had any history of trauma or sinusitis. All cases responded poorly to intravenous antibiotics; however their symptoms improved with systemic steroids.

Scleritis may manifest as thickening of the eye wall, optic disc edema and ERD. Orbital cellulitis can also lead to optic neuritis. ERD is rare in orbital cellulitis but has been reported.^[13,14]^ We need to focus on the presence of a definite source of infection and sinusitis, response to intravenous antibiotics and use of systemic steroids, and their correlation with systemic autoimmune disease. This case included several elements: CT confirmed sinusitis. The patient complained of sinus symptoms. Elevated levels of infection markers. Ultrasound demonstrated the classic “T” sign. The symptoms improved with intravenous antibiotics and systemic steroids. Blood tests revealed elevated levels of CRP, ESR, and white blood cell. While these lab abnormalities align with infection, they are nonspecific and may also occur in isolated severe inflammatory conditions such as posterior scleritis. This makes differential diagnosis challenging. However, subsequent antibiotic treatment normalized infection markers, and the condition relapsed after discontinuing steroids without renewed elevation of infection indicators. These findings suggest a more probable diagnosis of intraorbital infection or systemic inflammation. Therefore, we preferred that orbital cellulitis and scleritis occur together or that orbital cellulitis induces posterior scleritis.

Erdöl et al observed a decrease in the amount of serous detachment on OCT in parallel with improvements in visual acuity.^[15]^ This was true in the present case. However, when the retina was fully apposed, the patient’s BCVA was 0.3 and did not return to her previous vision. The visual prognosis of this patient was poor and may have been due to a number of factors. Due to the long duration of the disease, persistent macular edema and subretinal exudates impaired photoreceptors.^[16]^ Studies have shown that delayed treatment with anti-vascular endothelial growth factor drugs reduces BCVA recovery in eyes with retinal vein occlusion and macular edema.^[17]^ In addition, the patient’s systemic steroids were interrupted during treatment. Studies have shown that scleritis recurs when the steroid dose is reduced too quickly to <0.5 mg/kg/24 hours (in about 69.2% of patients).^[12]^

The patient initiated broad-spectrum intravenous antibiotic therapy to address potential orbital pathogens in this case. Given the severity of visual impairment and clinical/imaging evidence of significant inflammation (posterior scleritis, optic neuropathy), methylprednisolone was administered intravenously. This decision was based on the acute and severe visual threat, primarily driven by an invasive inflammatory process. After initial improvement in vision and symptoms, the steroid dosage was gradually tapered. However, pain and erythema symptoms rapidly recurred after discontinuation. This recurrence during adequate antibiotic treatment was interpreted as strongly supporting the presence of a steroid-dependent inflammatory condition (such as posterior scleritis) as the primary driver of relapse, rather than uncontrolled infection. Consequently, steroid therapy was resumed with a longer, slower tapering regimen. However, the patient declined steroid treatment due to weight gain and switched to indomethacin for nonsteroidal anti-inflammatory therapy. In a previous case report, posterior scleritis was initially treated with oral prednisolone for 10 days, followed by 20 days of oral indomethacin. The symptoms improved and did not recur.^[15]^ Follow-up at discharge showed no recurrence, leading us to exclude the option of second-line immunosuppressants. Nonsteroidal drugs or immunosuppressants may be considered if the disease recurs or if the patient is intolerant to steroids, particularly in pediatric patients requiring long-term inflammation control to minimize steroid exposure. In this case, the patient responded well to nonsteroidal anti-inflammatory therapy (indomethacin) without recurrence, thus second-line immunosuppressants were not initiated.

Periorbital cellulitis can spread backward to the orbit, cavernous sinus, and brain and can therefore be fatal. An intense inflammatory response may mask changes in the wall of the eye, and multiple diseases may occur simultaneously. Therefore, patients presenting with signs of periorbital or orbital cellulitis should be immediately administered intravenous antibiotics. Early use of hormones reduces tissue edema and prevents deterioration. If scleritis is present, steroids should be continued and slowly tapered to prevent recurrence. Nonsteroidal drugs or immunosuppressants may be considered if the disease recurs or if the patient is intolerant to steroids.

## 4. Lessons from practice

The orbital CT scan did not suggest fungal infection (e.g., absence of bone destruction, characteristic soft tissue shadows) or abscess formation, and the child had neither prolonged medication use nor trauma. To preserve more vision during early treatment, steroids were administered. Although we judged early use of high-dose steroids in this patient to be safe in the absence of fungal infection based on clinical experience, risks may still exist. The relapse upon steroid withdrawal was a pivotal event, underscoring the persistent, steroid-dependent nature of the underlying scleritis and highlighting that the initial inflammatory process was not fully eradicated. This observation is crucial for clinicians, as it argues for a slower, more prolonged steroid taper in similar scenarios and prompts consideration of steroid or second-line immunosuppression in cases of repeated relapse.

## 5. Patient perspective on treatment

As with the patient in this case, I would like to share my experience with the treatment I received for orbital cellulitis and posterior scleritis. When I first experienced severe pain in my right eye and noticed a sudden loss of vision, I was very scared and worried about what might happen. The doctors quickly diagnosed my condition and started me on intravenous antibiotics and steroids. Initially, the treatment was intense, and I had to stay in the hospital for several days. However, I noticed that the pain and swelling began to improve within a few days, which gave me hope. After being discharged, I continued with oral steroids, but I experienced some side effects, such as weight gain, which made me hesitant to continue the medication. Unfortunately, my symptoms returned, and I had to return to the hospital. This time, the doctors adjusted my treatment and prescribed nonsteroidal anti-inflammatory drugs, which helped me manage my symptoms without the side effects I had experienced before. Over time, my vision gradually improved, although it did not return to normal. I am grateful to the medical team for their support throughout this challenging experience.

## Author contributions

**Data curation:** Lei Yang.

**Investigation:** Lei Yang.

**Supervision:** Shuangle Li.

**Writing – original draft:** Lei Yang.

**Writing – review & editing:** Shuangle Li.
